# Lemon Juice in Rheumatism

**Published:** 1854-01

**Authors:** G. Owen Rees


### *Remedial Effects of Lemon Juice in Rheumatism, and Sources of Fallacy connected with its Use.*

BY G. OWEN REES, M. D., F. R. S.

It is now nearly three years since I proposed to treat rheumatism by the administration of lemon juice. This plan having been greatly applauded by some, while it has been condemned by others, I feel myself called upon, as the proposer of the remedy, to offer a few remarks, which I trust may have the effect of in some measure reconciling those discrepancies in result that would seem to have prevailed up to the present time. The very striking and marked advantage which I derived from treating with lemon juice certain cases comprised under the generic term rheumatism, was a fact which, taken in connexion with its occasional failure, early produced, in my mind, a conviction that, under that term, we include diseases essentially different, and only similar in producing the same ultimate pathological results. In a short notice which appeared in *The Lancet* some months ago, I alluded to the probability of the above explanation applying to this difficulty, and my attention has since been directed to the determina- of those points of difference in rheumatic cases which might indicate to us those amenable to the lemon juice treatment, and those in which we cannot hope to observe its beneficial effects.

The first rheumatic cases in which I tried this remedy were of the acute kind, and I very soon discovered that the more chronic forms were not so readily relieved by the same treatment. Acute cases were, however, sometimes, though but rarely, obstinate in recovery. These resisted other remedies subsequently administered, and ran the usual course of several weeks, and though they seemed to prove that lemon juice had done as much good as anything else, they still left unexplained why acute cases, quite as severe, nay, far severer, had been relieved in a few days only. It is highly probabable that this difference in result may sometimes arise from causes we can scarcely hope to detect; but such instanees will probably be rare, and in most cases I believe the difficulty is to be cleared away.

Among the cases admitted from time to time into Guy's Hospital, I have had opportunities of satisfying myself that acute articular rheumatism is frequently complicated by the presence of three conditions, which tend to keep up the disease, and so to interfere with the favorable action of the lemon juice. These are—1stly, Syphilitic rheumatism. 2ndly, Gonorrhœal rheumatism. 3dly, A pseudo-rheumatic affection occurring in connexion with purulent discharges not necessairly gonorrhœal. Over these pathological conditions lemon juice exerts no favorable influence.

Now, not only may the above mentioned complications interfere with the success of the remedy, but I have seen it exhibited in the last described, where the whole of the rheumatism present might, in all probability, be correctly attributed to the existence of the purulent discharge from the vagina, and where no remedy whatever produced a favorable influence, till recourse was had to treatment directed to the cure of the discharge.

The true nature of cases such as these is very apt to be overlooked; and I shall therefore here give an instance showing the necessity of attending to the point in question:—

Ann B——, aged thirty, was admitted into Guy's Hospital, Oct. 13th, 1852, suffering from what appeared to be chronic rheumatic gout. When young she enjoyed good health, and commenced to menstruate when thirteen years old, and has always been regular. Has been married four years, and had a child two years after marriage. Soon after her confinement, she had an attack of rheumatism affecting the joints. The pains in the head, before complained of, and which had continued up to this time, now ceased. This attack rendered her incapable of performing her household duties, and it continued to the time of her admission.

State on admission : Joints of extremities much swollen, and very painful on pressure. There is copious yellow discharge from the vagina, but she denies ever having had gonorrhœa. Respiratory and heart sounds normal. Tongue clean, appetite good, bowels open. Pulse 84. Take iodide of potassium, three grains, in mint water, three times a day. Cod-liver oil night and morning.

18th.—Complains of hæmorrhoids ; much the same. Six leeches to the anus. Linseed poultice to the part affected.

25th.—No improvements in the pains in the joints. Guaiacum mixture, one ounce three times a day. Di-sulphate of quinine, two grains; acetic extract of colchicum, one grain ;powdered ipecacuanha, two grains ; mix, and make into pills ; to be taken night and morning.

29th.—Pains not at all relieved, but the medicine purges her. To discontinue the present medicines, and to take chalk mixture, one ounce, after each liquid stool, until diarrhœa cease.

Nov. 1st.—Pains no better. Diarrhœa soon left her, Lemon juice, three ounces, three times a day,

8th.—No better. The vaginal discharge still continues. Compound tincture of guaiacum, half a drachm; mucilage mixture, one ounce, three times a day. Dover's powder, eight grains every night.

11th.—Is no better. Soda poultice to the part affected. Continue medicine.

15th.—Is much the same, Benzoic acid, five grains ; compound tincture of champhor, half drachm ; mucilage mixture, one ounce, three times a day.

22d.—No improvement. Compound iodine julep, one ounce, three times a day. Dover's powder, five grains, every night. Turpentine linament.

Dec. 4th,—Is no better. Continue the medicine. From this date baths and iodine friction were tried up to.

Jan. 3d, 1853.—Shoulders and arms are now rather less painful. Pulse 78, full soft. Tongue clean. Bowels relieved once daily. Sleeps better at night. Conium mixture three times a day. Omit the baths.

13th.—Pains of limbs still continue. The discharge from the vagina is profuse. Powder of cubebs, one drachm, three times a day.

17th.—Is decidedly improved. The swelling and tenderness of joints less. The discharge from the vagina less. Bowels regular.

From the last date given, this case rapidly got well ; but as will be seen, neither lemon juice nor any other remedy afforded relief till the profuse purulent discharge was checked. The whole case was characterized by symptoms approaching to the subacute form ; and it therefore would not have been selected from what I already knew as one likely to be much benefited by lemon juice. The observations I have now made on the sequel of the case, will, however, I trust, be regarded as a step in advance, and serve to establish it as a more definite type of a class of cases which we shall in future at once exclude as unfit for treatment, either by lemon juice, or any other of the ordinary remedies employed against rheumatism, but easily relieved when their true pathology is ascertained.

From what I have advanced, it will be observed, that I consider lemon juice the antidote to the true rheumatic diathesis; and that I expect to account for failures in treatment, by detecting either complications, or complete pathological differences.

I have already given a case illustrative of the latter character, and I now wish in addition to direct attention to two other forms of disease belonging to the same class, which I have seen mistaken for rheumatism, and treated by lemon juice, without the least benefit being derived. The first of these is the pseudo-rheumatic affection sometimes observed during the course of Bright's disease. Though the pathology of this affection is as yet imperfectly understood, still the symptoms and progress differ sufficiently from those of true rheumatism to enable us to draw a distinction; and I believe we may now fairly add another distinctive mark in the fact that the disease in question is not benefited as true rheumatism is by the exhibition of lemon juice. I have seen the remedy here fall into temporary disrepute till the mistake was detected. The other form of disease I would allude to, and which is perhaps characterized by as severe tortue as any of those yet treated of, is observed in connexion with spinal deformity. Here the pains are more extreme, and vary in intensity according to the health of the individual. Of course lemon juice cannot here be expected to benefit the patient, though its credit as a remedy has often suffered owing to its administration in such pseudo-rheumatic cases.—*London Lancet.*

